# Eradication of bovine tuberculosis in Ireland: is it a case of now or never?

**DOI:** 10.1186/s13620-024-00282-z

**Published:** 2024-12-20

**Authors:** Simon J. More

**Affiliations:** Centre for Veterinary Epidemiology and Risk Analysis, School of Veterinary Medicine, UCD, University College Dublin, Belfield, Dublin, D04 W6F6 Ireland

**Keywords:** Bovine tuberculosis, Ireland,, Eradication, Constraints, Success

## Abstract

There has been a sharp disimprovement in the bovine tuberculosis (bTB) situation in Ireland in recent years. This commentary argues for critical programme change in three overarching themes relevant to the Irish bTB eradication programme, if eradication is to be successful: (1) *Limiting infection in cattle.* Residual (hidden) infection is an important constraint to eradication, due to the use of imperfect diagnostic tests. This is resolved with a risk-based approach, as is widely used in other national programmes, and would impact herd management, cattle trade and regionalisation. (2) *Limiting infection within and from wildlife*. Infection in wildlife is a key feature of bTB in many countries, including Ireland. Early research with badger vaccination has been promising. However, wide-scale badger vaccination has proved logistically challenging, and research to monitor progress is underway. It is unlikely that badger vaccination, in addition to current cattle controls, will be sufficient to achieve bTB eradication. (3) *Programme leadership, management, governance and cost-sharing.* There are a number of substantial, seemingly intractable, issues relating to programme leadership, management, governance and cost-sharing which alone are sufficient to preclude any sustained move to eradication. International examples of success are available, with funding models being critical to progress. In these three themes, most of the constraints are non-technical. If difficult decisions are not taken and the *status quo* is allowed to continue, there is a risk that infection may establish in further wildlife species, which may make eradication unattainable. Current decisions (including inaction) will impact future generations, including the general public (through the Exchequer) and future farming families.

## Background

A national programme to eradicate bovine tuberculosis (bTB, caused by infection of *Mycobacterium bovis*) has been in place in Ireland since the late 1950s. In recent years, the national bTB situation has disimproved substantially. As of 19 May 2024, the herd incidence was 5.06% (rising sharply from a record low of 3.37% in 2015). During the 12 months prior to 19 May 2024, 31,414 reactor animals were detected (compared to 15,317 throughout 2015). During 2023, the total programme costs were €108.4 million, of which €74 million was paid by the Exchequer.

These figures are worrying, and highlight the importance of ongoing, critical scrutiny of current approaches to bTB eradication in Ireland.

There is now a substantial body of scientific knowledge, from Ireland and more broadly, with respect to bTB, including its epidemiology and control. Further, lessons have been learned from those countries where bTB eradication has been successful or is moving towards a successful conclusion. This commentary argues for critical programme change in three overarching themes relevant to the Irish bTB eradication programme, if eradication is to be successful. These are the views of the author, who is drawing on knowledge and experience gained over many years as a veterinary epidemiologist in association with the Australian and Irish bTB eradication programmes.

### Limiting infection in cattle

Bovine tuberculosis is primarily a disease of cattle, and infection is primarily sustained in cattle populations as a result of cattle-to-cattle transmission and spillover from infected wildlife. In Ireland, cattle controls in the national programme have been guided by, and are in compliance with, relevant European Union (EU) legislation, including the detection of infected animals through annual testing with the single intradermal comparative tuberculin test (SICTT), and abattoir surveillance, and the restriction of known infected herds for a period, to limit onward spread of infection to other herds.

Despite these efforts, there is substantial evidence in support of residual (hidden) infection as an important constraint to eradication, whereby infected – but undetected – animals remain in their herd of origin or are moved to another herd. This body of evidence has been gathered using a range of disciplinary approaches, including classical epidemiology (including [21]), whole genome sequencing (including [1]), and computer modelling (including [7]). Existing diagnostic tests are imperfect, and there is the potential to miss a substantial number of infected animals. This includes both the SICTT, which is used as a screening test, and the follow-up interferon-γ test, which is a higher sensitivity test used in known infected herds. Residual infection poses a risk to the herd in which these animals are present, in limiting our ability to ‘clear’ these herds of infection. They also pose a risk to purchasing herds, given the potential that animals with residual infection might move – undetected – from one farm to another. This concern is exacerbated by the very high levels of cattle movement in Ireland [15]. Over many years, there has been ongoing work internationally towards improved diagnostic tests, but without substantial success to date.

There is a technical solution to the problem of residual infection (and imperfect testing), which has been widely adopted elsewhere over many decades. This solution, known as a ‘risk-based approach’, was central throughout the successful eradication programme in Australia (since 1970, [17]), and is also central to the ongoing bTB eradication programme in New Zealand [19] and the United States of America (USA, [18]). This approach is also used with other diseases of farmed animals, such as Johne’s disease, where imperfect testing is also a challenge. Using this approach, there is a shift in thinking from a ‘black and white’ perspective (that is, a herd is infected or it is not) through to a more nuanced perspective of ‘shades of grey’ (herds are placed at a point on a risk gradient, from high risk through to low risk, depending on the likelihood that the herd is infected). The following are examples of risk-based approaches within a national bTB eradication programme:

- *Herd management.* Immediately after a herd is derestricted (which generally occurs once two clear full-herd tests are achieved), it would be considered at high risk, particularly following a large breakdown (that is, there is a high risk that infected animals are still present). Over time and with successive negative full-herd tests, assurance is being built that infection has been eliminated from the herd. Therefore, the herd will progressively move to medium risk, then to low risk.
- *Cattle trading rules.* A risk-based approach to trading (‘risk-based trading’) is used to limit the potential that infection might spread when animals are bought and sold. Using this approach, trading rules are established to require purchased cattle to be derived from herds of equivalent or lower risk, and animals that are consequently sold to move solely to herds of equivalent or higher risk.
- *Regionalisation.* Distinct geographical areas are delineated to allow disease control to be differentiated at area level based on infection risk, to allow resource allocation to be prioritised and lower risk areas to be protected. Regionalisation is a key tool in national bTB control/eradication programmes, and has been applied in all countries where eradication has been achieved or is progressing [12].

It has not yet been possible to introduce a comprehensive risk-based approach to bTB control in Ireland. Since at least the early 1990s, farm organisations have strongly opposed their introduction, in large part due to the potential devaluing of cattle from herds deemed at high(er) risk. In Australia, the impact of risk-based approaches in that country were essentially positive, contributing to fundamental changes in national cattle trade and substantial improvements to cattle management in northern parts of the country. A range of initiatives were implemented, including assigning risk at the level of the group (not the individual), a dynamic system of risk-based herd and area classification, restrictions on the movement of cattle between herds and areas on the basis of herd and area risk, and a broad range of strategies to effectively manage residual bTB risk [9, 17].

### Limiting infection within and from wildlife

In Ireland, as in many countries internationally, *M. bovis* has established in wildlife populations. Here, infection is maintained in badger populations throughout the country, and in deer populations in Co. Wicklow, spilling back to cattle on occasions. In New Zealand, the primary wildlife host is brushtail possum, whereas it is white-tailed deer in parts of the USA, and a range of wildlife species, including lion and buffalo, in parts of southern Africa.

Focused badger culling has been conducted in Ireland since 2004 [5], as an interim measure to limit transmission from wildlife-to-cattle. However, vaccination is preferred in the long-term, noting that badgers are a protected national species. Based on detailed research, both experimental and in the field, vaccination has been confirmed as a viable control option, and vaccination would be ‘no worse than’ culling in controlling bTB in cattle [14].

Since 2018, substantial areas of the country have now been ‘turned over’ from culling to vaccination. Experience since this time has highlighted the logistical challenges with wide-scale badger vaccination, including difficulties in achieving sustained levels of vaccination coverage (this being the overall proportion of the badger population in a region, at any point in time, that have been vaccinated). Results have been variable, and focused culling has been re-introduced in some of these areas following bTB outbreaks in cattle. This has raised two critical scientific questions for all interested parties, including whether badger vaccination is working, and, if it is, whether it will be sufficient in addition to current cattle controls to effectively lead to bTB eradication.

The question as to whether badger vaccination is working is best answered by considering both vaccine efficacy (the proportion of animals that are protected following vaccination) (but noting that vaccination does not protect badgers when delivered post-infection, [10]), and the effectiveness of the overall vaccination programme (which considers both vaccine efficacy and vaccination coverage). During a large field trial in Co. Kilkenny during 2009–13, vaccine efficacy of 59% was estimated [3]. Estimates of vaccination coverage are not yet available, and will be influenced both by the number of badgers vaccinated and aspects of badger ecology including badger lifespan. A large Irish dataset is currently being analysed, based on data collected from badgers across 9 vaccine areas during 2020-23, which will provide field-based estimates of vaccine efficacy, vaccination coverage, and the effectiveness of the overall vaccination programme over recent years. These data are also being used to evaluate the performance of field diagnostic tests, which are undertaken prior to vaccination to determine whether badgers are already infected (in such circumstances, we know that the vaccine will not work).

Several modelling approaches have been used to determine whether badger vaccination, in addition to current cattle controls, will be sufficient to achieve bTB eradication. An initial – and simpler – model suggested that badger vaccination, in addition to current cattle controls, should be sufficient, but only just [2]. That is, the reproduction number (R_0_) of the cattle-badger system would be reduced, at best, to just below 1. However, the model did not account for several uncertainties, and the results may have been over-optimistic. Regardless, time to eradication would be very long, and possibly many decades, if R_0_ were just below 1, and there was a clear recommendation that further cattle controls would be needed. The second model was more comprehensive as it considered both the spatial variation in infection dynamics within a region, and the linkages (through cattle trade) between individual farms [6]. These results are less optimistic than that of Aznar et al. [3], and suggest that bTB eradication cannot be achieved through badger vaccination alone, in addition to existing cattle controls. In approximately 30% of farms, R_0_ will not be reduced below 1, and infection would be maintained in a region. The work has shown that eradication is only possible if multiple transmission routes are simultaneously controlled.

It will take some time before the results of current research are available. There will be an ongoing need both to limit the spill-back of infection from badgers to cattle , usingmethods that are acceptable to the Irish public and consistent with Ireland’s international obligations, and to gain experience with the implementation and evaluation of the vaccination programme, building on existing work. In light of current challenges to vaccination roll-out, it would be prudent to consider regionalisation of effort, to allow focused use of finite resources [12].

### Programme leadership, management, governance and cost-sharing

In the early 1970s in Australia and New Zealand, there was a ‘burning platform’ (ie the need for drastic action to avoid a catastrophic outcome) for national bTB eradication, given the fears at that time of the long-term security of key international markets for agricultural products if bTB were not eradicated [17]. In Ireland, there is currently no such burning platform, in part due to the protection gained from the EU internal market. However, this may well change considering the increasing exposure of Irish agricultural products to international trade. In 2023, markets outside the EU accounted for 65% of the overall annual value of €6.5 billion in dairy product exports and 52% of the overall annual value of €2.7 billion in beef exports (Bord Bia [4]). These markets could reasonably become sensitive to bTB concerns, particularly if bTB risk were to persist or increase, and (perceived) alternatives at equivalent quality but lower risk were available.

There are a number of substantial, seemingly intractable, issues relating to programme leadership, management, governance and cost-sharing:

- There is a fundamental – and ongoing – mismatch between responsibility, costs and benefits across key programme partners. The government essentially carries all responsibility (bTB eradication is seen by most as a responsibility of government), is the majority funder, but a minority beneficiary (22% of all benefits, primarily increased tax returns arising from increased market access). In contrast, farmers carry little responsibility for the national programme, are a minority funder, and the main beneficiary (78% of all benefits, primarily market access) (Grant Thornton [11]).
- The primary programme beneficiaries (farmers) have been in long-term conflict with the government about the programme. The government have also taken most – if not all – of the criticism for any failure in programme progress, both publicly and in private.
- As outlined throughout this article, there are a number of critical decisions that have not been made, but are essential if eradication is to be achieved.
- Overall farmer contributions to the programme are relatively insensitive to the overall national bTB situation. The farmer contribution includes the cost of an annual herd test, disease levies and a contribution to income supplement (it is only the latter that is linked to national bTB incidence). Therefore, the financial impact from progress (or disimprovement) in the national programme progress is limited for the many Irish farmers who do not suffer a bTB outbreak in their herd.
- The programme is very susceptible to political pressure, and the focus of intensive lobbying by farm organisations. There are ongoing demands for (additional) funding and programme exceptions, noting that each exception has the potential to prolong the overall time to eradication.
- In common with other areas of public discourse, values are increasingly more influential that facts in shaping public opinion. Within the national bTB discussion, an understanding of, and commitment to, science and evidence-based decision-making is under ongoing challenge [16].

These issues alone are sufficient to preclude any sustained move towards eradication.

The experiences of Australia and New Zealand are instructive, as programme success in each of these countries has been attributed, in part, to fundamental changes to leadership, management, governance and cost-sharing that developed in response to pivotal political decisions [20]. To illustrate:

- The Australia programme was initially government-run, and it was only as a consequence of substantial industry unrest in 1984 (some 14 years after programme start) that the decision was made by the relevant Federal Minister for major programme reform, including a sharing of responsibilities and costs by government and the farming industry from that point forward. This model now underpins the governance of animal health programmes in that country.
- In New Zealand, bTB eradication was undertaken in the context of major economic reforms in the 1980s, including market deregulation and a removal of agricultural subsidies. The bTB programme was managed as a government-industry partnership from 1989 [20], and since 2013 as an independent organisation solely responsible and accountable for developing and implementing government and industry-approved strategies and plans for controlling bTB in New Zealand [13]. The cost-sharing model – legally agreed and in compliance with national legislation – is based on formal recognition of beneficiaries (those who benefit from bTB control) and exacerbators (those who exacerbate the problem, either through their action or inaction).

In both countries, funding models have been critical to progress. Indeed, it is this approach that has provided a burning platform for ongoing industry commitment. Tweddle and Livingstone [20] note that the involvement of industry in both funding and policy development has contributed to the commitment of industry, and through peer pressure, of individual farmers to the programmes. In both countries, a levy has been used to collect programme funding from primary producers.

## Conclusions

Ireland will not be able to eradicate bTB given the current trajectory of infection in the national herd. In two of the three above-mentioned areas of concern (cattle controls; programme leadership, management and governance), current approaches are not aligned with international best-practice. Further, in these two areas of concern, knowledge gaps are not a fundamental constraint to progress. In the third area of concern, relating to wildlife, some important technical aspects remain unresolved, and further research will be needed. Nonetheless, there are options available, particularly the use of regionalisation to enable available resources to be prioritised, which would greatly assist.

There are several key concerns if difficult decisions are not taken and the *status quo* is allowed to continue:

- From a biological perspective, there is a substantial risk associated with the recent national resurgence of infection. In particular, there is a risk that infection may establish in further wildlife maintenance hosts, beyond badgers nationally and deer in Co. Wicklow. Eradication may become unattainable if infection were to establish in additional wildlife hosts, including deer populations in areas outside Co. Wicklow.
- From a societal perspective, there will only be losers if difficult decisions continue to be ‘kicked down the road’. Rather, if current controls are insufficient, this will substantially increase both the time to (eventual) eradication and overall programme costs. Remember that the costs of this programme are very substantial, perhaps €2 billion every 20 years. Current decisions (including inaction) will impact future generations, including the general public (through the Exchequer) and future farming families.

There is one possible alternative to bTB eradication, namely bTB suppression, but it is far from attractive. With suppression, bTB levels in the national cattle herd would need to be kept at or below levels deemed acceptable to international buyers. Here, we would need to remain competitive with other major agrifood exporting countries, including those where bTB has essentially been eradicated (such as the Netherlands) or is under very good control in the cattle population (New Zealand, with 19 known infected cattle and deer herds in mid 2023, compared with 5,172 infected cattle herds in Ireland during 2023) [19, 8]. The cost of bTB suppression each year will be very substantial, given that the current annual spend of €108.4 million (the programme cost in 2023) has not yet been successful in reversing a progressively worsening national situation. Because there is no attempt to eradicate infection, the costs of bTB suppression will continue in perpetuity.

## Data Availability

Not applicable.
